# Supplementary material for: Gestational Weight Gain—Re-Examining the Current Paradigm
Source: Nutrients. 2020 Aug 1;12(8):2314. doi: 10.3390/nu12082314 (PMC7468983; doi:10.3390/nu12082314)

**SUPPLEMENTARY TABLES**

**Supplementary Table 1: Relative Risk of Excess gestational weight gain by Dietary Characteristics and Physical Activity in LIMIT and OPTIMISE Standard Care Group.**

Estimates are relative risks (RR) of excess gestational weight gain (GWG) (95% CI) corresponding to the stipulated increase in dietary intake, or physical activity, at each time point.

| **Characteristic** | **LIMIT Estimate (95% CI)** | **LIMIT p** | **OPTIMISE Estimate (95% CI)** | **OPTIMISE p** |
| --- | --- | --- | --- | --- |
| Energy (kJ): +100^b^ |  | 0.012* |  | 0.467* |
| - BL | 1.00 (0.99, 1.00) | 0.431 | 0.99 (0.97, 1.01) | 0.276 |
| - 28w | 0.99 (0.99, 1.00) | 0.023 | 1.01 (0.99, 1.02) | 0.255 |
| - 36w | 1.01 (1.00, 1.01) | 0.002 | 1.00 (1.00, 1.01) | 0.720 |
| Carbohydrate (g): +10^b^ |  | 0.007* |  | 0.575* |
| - BL | 0.99 (0.98, 1.01) | 0.293 | 0.98 (0.92, 1.05) | 0.595 |
| - 28w | 0.99 (0.97, 1.00) | 0.048 | 1.02 (0.97, 1.08) | 0.423 |
| - 36w | 1.02 (1.01, 1.03) | 0.001 | 1.01 (1.01, 1.03) | 0.598 |
| Fibre (g): +10^a^ |  | 0.254* |  | 0.675* |
| - BL | 1.03 (0.94, 1.13) | 0.533 | 0.89 (0.59, 1.32) | 0.551 |
| - 28w | 0.93 (0.84, 1.04) | 0.225 | 1.33 (0.83, 2.13) | 0.234 |
| - 36w | 1.10 (0.99, 1.22) | 0.087 | 0.85 (0.99, 1.22) | 0.510 |
| Total Fat (g): +10^a^ |  | 0.041* |  | 0.314* |
| - BL | 0.96 (0.91, 1.01) | 0.128 | 0.92 (0.79, 1.07) | 0.282 |
| - 28w | 0.95 (0.90, 1.01) | 0.122 | 1.08 (0.91, 1.30) | 0.383 |
| - 36w | 1.07 (1.02, 1.13) | 0.011 | 0.91 (1.02, 1.13) | 0.299 |
| Protein (g): +10^b^ |  | 0.028* |  | 0.091* |
| - BL | 1.00 (0.96, 1.04) | 0.919 | 0.97 (0.86, 1.10) | 0.674 |
| - 28w | 0.94 (0.90, 0.98) | 0.008 | 1.07 (0.95, 1.21) | 0.276 |
| - 36w | 1.06 (1.01, 1.10) | 0.009 | 0.86 (1.01, 1.10) | 0.039 |
| Sugars (g): +10^b^ |  | 0.117* |  | 0.429* |
| - BL | 1.00 (0.97, 1.03) | 0.987 | 1.02 (0.94, 1.11) | 0.649 |
| - 28w | 0.99 (0.96, 1.01) | 0.305 | 1.04 (0.94, 1.14) | 0.452 |
| - 36w | 1.03 (1.00, 1.05) | 0.022 | 1.01 (1.00, 1.05) | 0.859 |
| HEI: +10^c^ |  | 0.781* |  | 0.984* |
| - BL | 1.05 (0.88, 1.26) | 0.562 | 0.92 (0.57, 1.48) | 0.720 |
| - 28w | 0.93 (0.78, 1.09) | 0.366 | 1.00 (0.52, 1.91) | 0.998 |
| - 36w | 1.04 (0.89, 1.21) | 0.618 | 1.02 (0.89, 1.21) | 0.926 |
| METs: +1000^c^ |  | 0.859* |  | 0.181* |
| - BL | 0.99 (0.96, 1.02) | 0.430 | 1.06 (0.98, 1.15) | 0.117 |
| - 28w | 1.00 (0.96, 1.03) | 0.859 | 0.97 (0.86, 1.09) | 0.587 |
| - 36w | 1.01 (0.97, 1.04) | 0.737 | 0.92 (0.97, 1.04) | 0.172 |

^a^ Relationships between diet/physical activity and excess GWG for both LIMIT and OPTIMISE data were modelled using log binomial regression. Estimates are Relative Risks (95% CI), adjusted for prepregnancy BMI (continuous), parity (0 vs 1+), age at trial entry, smoking status and SEIFA IRSD quintile.

^b^ Relationships between diet/physical activity and excess GWG for both LIMIT and OPTIMISE data were modelled using log Poisson regression with robust variance, due to nonconvergence of log binomial models.

^c^ Log binomial regression was used for LIMIT data, but log Poisson models with robust variance estimation were used for OPTIMISE data, due to nonconvergence of the log binomial model.

* denotes p value for global test of any relationship between diet/physical activity (across all time points) and risk of excess GWG.

**Supplementary Table 2:** **Gestational weight gain and relationship with birth outcomes.**

The table below shows the proportions of excess GWG in each 2-unit BMI group, alongside the average total GWG, proportion of large for gestational (LGA), caesarean section, and the mean birthweight z-score. The relationships between excess GWG and outcomes are also shown in the graphs.

| **BMI Group** | **Excess GWG kg** | **Total GWG kg** | **Large for gestational age** | **Birthweight z score** | **Caesarean Section** |
| --- | --- | --- | --- | --- | --- |
| 19-20.9 | 0.08 (0.02, 0.15) | 11.21 (10.41, 12.00) | 0.07 (0.01, 0.13) | -0.14 (-0.35, 0.08) | 0.28 (0.18, 0.39) |
| 21-22.9 | 0.11 (0.05, 0.17) | 11.53 (10.86, 12.19) | 0.06 (0.02, 0.11) | -0.03 (-0.20, 0.14) | 0.22 (0.14, 0.30) |
| 23-24.9 | 0.18 (0.11, 0.25) | 12.25 (11.52, 12.98) | 0.12 (0.06, 0.19) | 0.21 (0.04, 0.38) | 0.22 (0.14, 0.30) |
| 25-26.9 | 0.51 (0.43, 0.59) | 11.71 (11.03, 12.39) | 0.14 (0.09, 0.19) | 0.30 (0.15, 0.44) | 0.29 (0.22, 0.36) |
| 27-28.9 | 0.41 (0.34, 0.49) | 10.93 (10.15, 11.71) | 0.20 (0.14, 0.26) | 0.40 (0.23, 0.57) | 0.34 (0.26, 0.41) |
| 29-30.9 | 0.45 (0.36, 0.54) | 10.38 (9.44, 11.32) | 0.18 (0.11, 0.24) | 0.42 (0.23, 0.62) | 0.29 (0.20, 0.37) |
| 31-32.9 | 0.54 (0.44, 0.63) | 9.34 (8.20, 10.47) | 0.20 (0.13, 0.28) | 0.26 (0.04, 0.47) | 0.35 (0.26, 0.44) |
| 33-34.9 | 0.38 (0.28, 0.49) | 8.37 (7.16, 9.59) | 0.20 (0.11, 0.28) | 0.51 (0.29, 0.73) | 0.37 (0.27, 0.47) |
| 35-36.9 | 0.39 (0.27, 0.51) | 8.69 (7.20, 10.19) | 0.20 (0.10, 0.30) | 0.40 (0.16, 0.64) | 0.42 (0.30, 0.54) |
| 37-38.9 | 0.35 (0.22, 0.48) | 6.49 (4.87, 8.11) | 0.25 (0.14, 0.37) | 0.65 (0.37, 0.92) | 0.37 (0.24, 0.51) |
| 39-40.9 | 0.30 (0.15, 0.44) | 6.14 (4.17, 8.11) | 0.30 (0.15, 0.44) | 0.39 (-0.00, 0.78) | 0.43 (0.27, 0.59) |

GWG gestational weight gain

Numbers for total GWG and birthweight zscore are mean (se of mean), and those for Excess GWG, LGA, Csection are proportion (standard error).

**Supplementary Figure 1: Gestational weight gain across BMI and proportions of selected outcomes.**


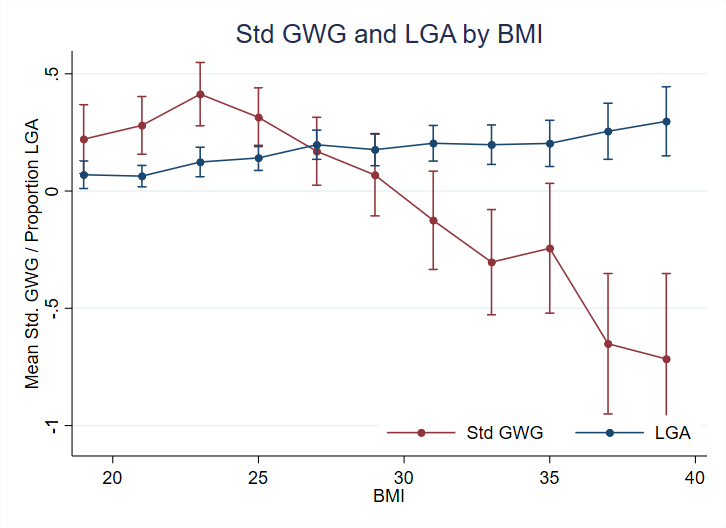

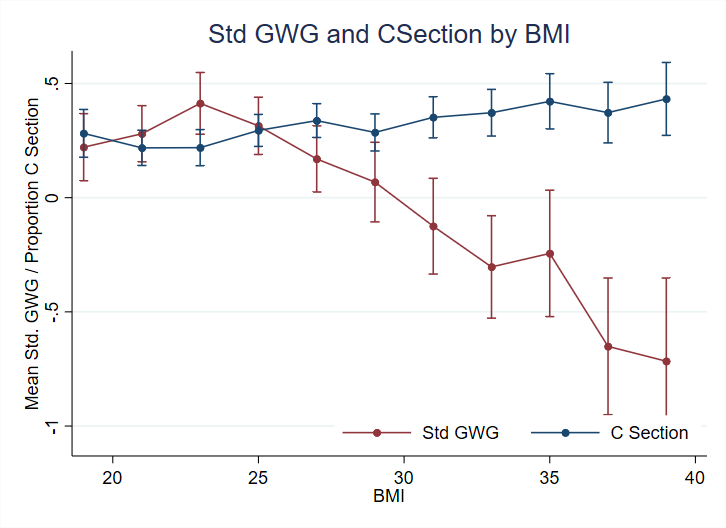


Supplementary figures 1-3 illustrate the relationship between pre-pregnancy BMI (grouped into 2 kg/m^2^ categories, for visualisation purposes) and GWG (standardized as number and standard deviation (SD) from the mean) in the control groups from all three studies along with a) proportions of LGA infants, b) proportion of caesarean deliveries, and c) birth weight z-score. The proportion of women with excess GWG decreases as BMI increases, whilst proportion of LGA, caesarean birth and birth weight z-score increase.

**Figure 1a**: Total GWG and Proportion of LGA Infants, by BMI

**Figure 1b**: Total GWG, and Proportion of Caesarean Deliveries, by BMI

**Figure 1c:** Total GWG and mean birthweight z-score, by BMI


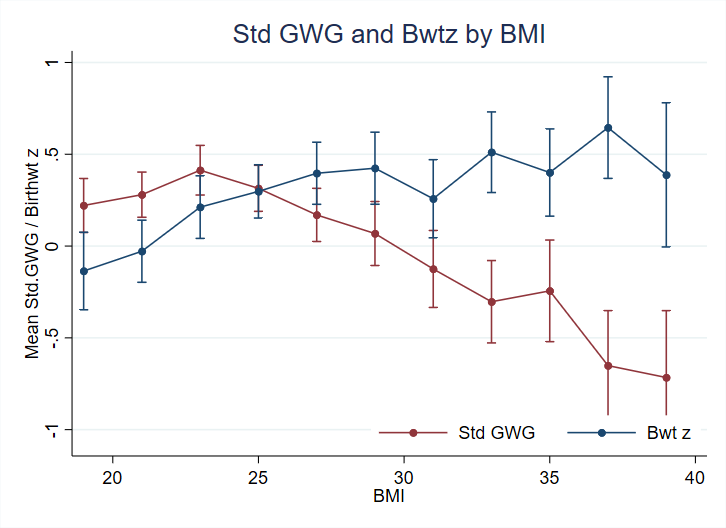

Supplement: Supplementary file 1 [file nutrients-12-02314-s001.zip › nutrients-883802-supplementary.docx]
